# Supplementary material for: Fungal community composition predicts forest carbon storage at a continental scale
Source: Nat Commun. 2024 Mar 16;15:2385. doi: 10.1038/s41467-024-46792-w (PMC10944544; doi:10.1038/s41467-024-46792-w)
Supplement: Supplementary file 1 — Supplementary Information [file 41467_2024_46792_MOESM1_ESM.pdf]

Supplementary Information

Fungal community composition predicts forest carbon storage at a continental scale

Mark A. Anthony[1,2,3,*], Leho Tedersoo[4], Bruno De Vos[5], Luc Croisé[6], Henning Meesenburg[7], Markus Wagner[7], Henning Andreae[8], Frank Jacob[8], Paweł Lech[9], Anna Kowalska[9], Martin Greve[10], Genoveva Popova[11], Beat Frey[2], Arthur Gessler[1,2], Marcus Schaub[2], Marco Ferretti[2], Peter Waldner[2], Vicent Calatayud[12], Roberto Canullo[13], Giancarlo Papitto[14], Aleksander Marinšek[15], Morten Ingerslev[16], Lars Vesterdal[16], Pasi Rautio[17], Helge Meissner[18], Volkmar Timmermann[19], Mike Dettwiler[1], Nadine Eickenscheidt[20], Andreas Schmitz[20, 21], Nina van Tiel[1,22], Thomas W. Crowther[1], Colin Averill[1]

[1]Department of Environmental Systems Science, ETH Zürich, Zürich, Switzerland
[2]Swiss Federal Institute for Forests, Snow, and the Landscape Research (WSL), Birmensdorf, Switzerland
[3]Center for Microbiology and Environmental Systems Science, University of Vienna, Vienna, Austria.
[4]Mycology and Microbiology Center, University of Tartu, Tartu, Estonia
[5]Environment & Climate Unit, Research Institute for Nature and Forest, Geraardsbergen, Belgium
[6]French National Forest Office, Fontainebleau, France
[7]Northwest German Forest Research Institute, Göttingen, Germany
[8]Sachsenforst State Forest, Pirna OT Graupa, Germany
[9]Forest Research Institute, Sękocin Stary, Poland
[10]Research Institute for Forest Ecology and Forestry, Trippstadt, Germany
[11]Executive Environmental Agency at the Ministry of Environment and Water, Sofia, Bulgaria
[12]Mediterranean Center for Environmental Studies, Paterna, Spain
[13]Department of Plant Diversity and Ecosystem Management, University of Camerino, Camerino, Italy
[14]Arma dei Carabinieri Forestry Environmental and Agri-food protection Units, Rome, Italy
[15]Slovenian Forestry Institute, Ljubljana, Slovenia
[16]Department of Geosciences and Natural Resource Management, University of Copenhagen, Frederiksberg C, Denmark
[17]Natural Resources Institute Finland, Rovaniemi, Finland
[18]Division of Forest and Forest Resources, Norwegian Institute of Bioeconomy Research, Ås, Norway
[19]Division of Biotechnology and Plant Health, Norwegian Institute of Bioeconomy Research, Ås, Norway
[20]State Agency for Nature, Environment and Consumer Protection of North Rhine-Westphalia, Recklinghausen, Germany
[21]Thuenen Institut of Forest Ecosystems, 16225 Eberswalde, Germany
[22]Environmetnal Computational Science and Earth Observation Laboratory, EPFL, Lausanne, Switzerland
[*]Correspondence should be addressed to Mark A. Anthony (manthony5955@gmail.com)

Supplementary Table 1. Statistical summaries comparing models of tree growth with fungal versus bacterial species richness (# of operational taxonomic units; OTUs) as predictor variables. Data shows the results of generalized additive modeling, including model estimates (i.e., coefficients), standard errors (SE), and *P*-values from full statistical models including all co-variables. Note that each model contains only one microbiome predictor.

**Parametric terms**	Estimate	SE	*t*-value	*P*-value
(Intercept)	-2.6573108	2.9009066	-0.916	0.36387
Fungal richness	0.5111837	0.2304736	2.218	0.03094
N deposition	0.1259197	0.364195	0.346	0.73092
Mineral Nitrogen Stock	0.4918893	0.1429244	3.442	0.00115
MAT	0.000856	0.0061821	0.138	8.90E-01
MAP	-0.000385	0.0004432	-0.869	3.89E-01
Soil pH	-0.1918281	0.1133522	-1.692	0.09656
Soil clay content	-0.0072983	0.0194758	-0.375	0.70938
Functional tree type	-0.7137729	0.242046	-2.949	0.00477
Stand age	0.0073393	0.0042508	1.727	0.09018
**Smooth term**	Est. DF	Ref. DF	*F*-value	*P*-value
Stem density	1.918	1.993	18.91	1.59E-06
**Parametric terms**	Estimate	SE	*t*-value	*P*-value
(Intercept)	-1.33E+01	4.14E+00	-3.198	0.00256
Bacterial richness	1.04E+00	5.24E-01	1.983	0.05363
N deposition	7.89E-01	3.12E-01	2.531	0.01503
Mineral Nitrogen Stock	3.79E-01	1.30E-01	2.907	0.0057
MAT	-1.15E-02	6.62E-03	-1.744	0.08807
MAP	-4.28E-04	5.36E-04	-0.798	0.42891
Soil pH	7.73E-02	1.13E-01	0.683	0.49821
Soil clay content	1.02E-02	2.02E-02	0.505	0.6158
Functional tree type	-3.77E-01	2.21E-01	-1.706	0.09514
Stand age	8.78E-03	4.03E-03	2.177	0.03491
**Smooth term**	Est. DF	Ref. DF	*F*-value	*P*-value
Stem density	1.966	1.999	15.93	7.68E-06

Supplementary Table 2. Statistical summaries of models of tree growth with fungal composition (PCoA1 and PCoA2) in the organic horizon. Predictors included PCoA axes for total fungi. Data shows the results of generalized additive modeling, including model estimates (i.e., coefficients), standard errors (SE), and *P*-values from full statistical models including all co-variables. Note that each model contains only one microbiome predictor.

	Estimate	SE	*t*-value	*P*-value
(Intercept)	1.2344238	2.722149	0.453	0.6521
Fungal PCoA1	-1.6202941	0.962764	-1.683	0.0985
N deposition	-0.0033244	0.4283468	-0.008	0.9938
Organic N stock	-0.1031805	0.0898635	-1.148	0.2562
MAT	0.0064607	0.0083623	0.773	0.4433
MAP	0.000696	0.0004715	1.476	1.46E-01
Soil pH	-0.320192	0.128201	-2.498	1.58E-02
Soil clay content	-0.0176682	0.0207586	-0.851	0.3987
Functional tree type	-0.8488874	0.2546885	-3.333	0.0016
Stand age	0.011126	0.0051554	2.158	0.0356
Smooth term	Est. DF	Ref. DF	F-value	P-value
Stem density	1.811	1.964	15.33	1.42E-05
	Estimate	SE	*t*-value	*P*-value
(Intercept)	-0.985611	2.4867703	-0.396	0.6935
Fungal PCoA2	-2.8524385	1.0723338	-2.66	0.01041
N deposition	0.4128144	0.3980336	1.037	0.30455
Organic N stock	-0.0119976	0.0921148	-0.13	0.89688
MAT	0.0023515	0.0078527	0.299	0.76581
MAP	0.0004503	0.000466	0.966	3.39E-01
Soil pH	-0.3668078	0.1238869	-2.961	4.64E-03
Soil clay content	-0.0244192	0.0203232	-1.202	0.23508
Functional tree type	-0.8019711	0.2451975	-3.271	0.00192
Stand age	0.0122024	0.0048105	2.537	0.01429
Smooth term	Est. DF	Ref. DF	F-value	P-value
Stem density	1.88	1.985	18.09	1.94E-06

Supplementary Table 3. The relative abundance of fungal functional group annotations. Italicized groups are those within the group above (e.g., wood saprotroph are a group within the saprotrophs annotated as “wood saprotrophs” in FUNGuild). Values represent the mean relative abundance across all samples.

Soil horizon	Group	Relative abundance (%)
Organic	Ectomycorrhizal	23.8
	‘Pure' ectomycorrhizal	19.3
	Saprotroph	38.5
	‘Pure' saprotroph	15.8
	Woot saprotroph	14.2
	Endophyte	13.5
	Plant pathogen	7.3
	Ericoid mycorrhizal	2.6
Mineral	Ectomycorrhizal	26.4
	‘Pure' ectomycorrhizal	22.7
	Saprotroph	33.6
	‘Pure' saprotroph	15.5
	Wood saprotroph	8.9
	Endophyte	11.2
	Plant pathogen	3.8
	Ericoid mycorrhizal	3.5

Supplementary Table 4. Statistical summaries comparing models of tree growth with different fungal functional group richness predictor variables. Data shows the model estimates (coefficients), standard errors (SE), and *P*-values from full statistical models including all co-variables. We only show the results for microbiome model output for brevity because other co-variable effects are shown in Table S1. Note that each model contained only one microbiome predictor. Models with ‘pure’ ectomycorrhizal fungal (Ectomyco.) and saprotroph richness are those which were exclusively assigned to one versus multiple functional guilds (e.g., saprotroph-pathogen). Note that pure category models were not made for endophytes nor pathogens because it is expected that these groups will have more than one trophic mode.

	**Estimate**	**SE**	***P*-value**
Ectomyco. richness	0.04590836	0.1492676	0.7596
Endophyte richness	0.67559496	0.2333078	0.0055
Plant pathogen richness	0.41004186	0.195254	0.04059
Wood saprotroph richness	0.37284712	0.2035421	0.0727
Ericoid richness	4.68E-01	0.1973914	0.02168
Saprotroph richness	0.50018564	0.2269	3.19E-02
“Pure” ectomyco. richness	0.02906632	0.1470056	0.8440
“Pure” saprotroph richness	0.55919943	0.2279858	0.0176

Supplementary Table 5. Eigenvalues for principal coordinate analysis axes 1 and 2 for all microbial groups considered in the study.

**Group**	**Axis**	**Eigenvalue**
Bacteria	PCoA1	25
Bacteria	PCoA2	11
Fungi	PCoA1	11
Fungi	PCoA2	9
Ectomycorrhizal	PCoA1	9
Ectomycorrhizal	PCoA2	8
Pure' ectomycorrhizal	PCoA1	10
Pure' ectomycorrhizal	PCoA2	9
Saprotroph	PCoA1	13
Saprotroph	PCoA2	11
Pure' saprotroph	PCoA1	21
Pure' saprotroph	PCoA2	9
Endophytes	PCoA1	17
Endophytes	PCoA2	14
Wood saprotroph	PCoA1	20
Wood saprotroph	PCoA2	11
Ericoid mycorrhizal	PCoA1	4
Ericoid mycorrhizal	PCoA2	3
Plant pathogen	PCoA1	13
Plant pathogen	PCoA2	11

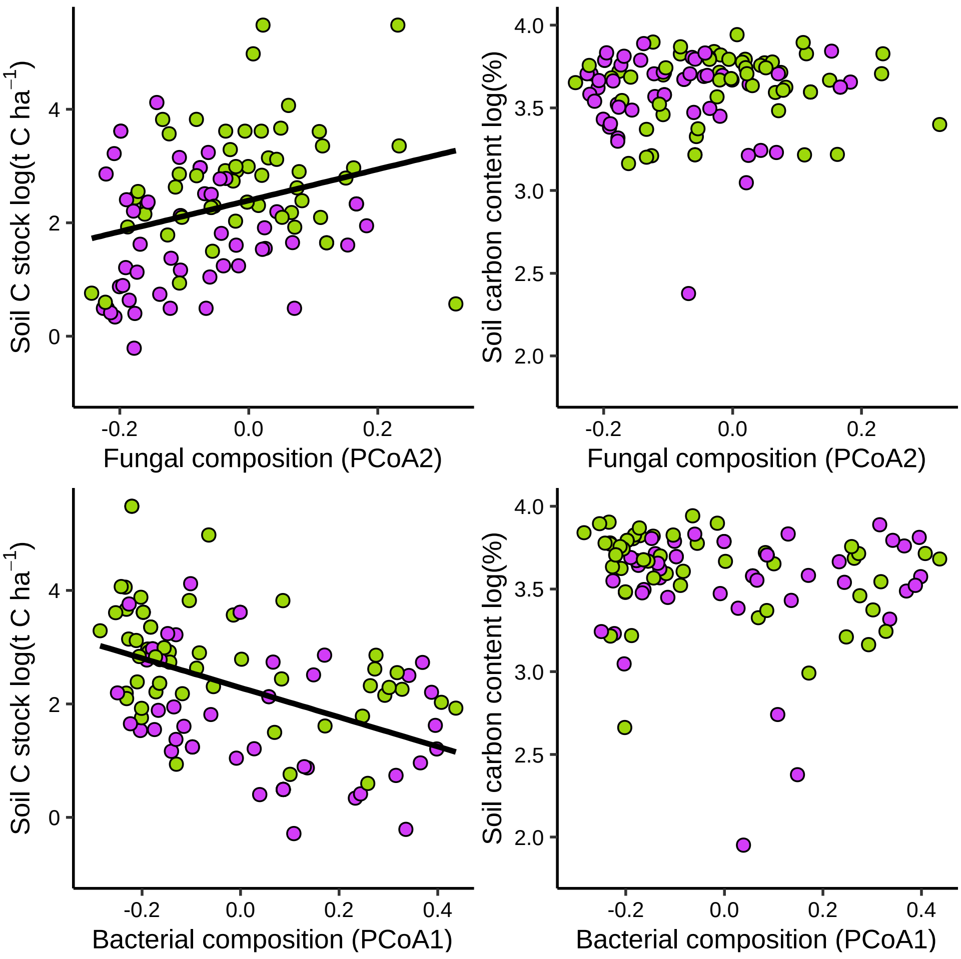

Supplementary Fig. 1. Scatterplots showing that microbiome composition is only correlated with soil carbon (C) stocks, not soil carbon content. Green and purple points show conifers and broadleaves, respectively. See Figure 4 in the main text for statistical overview of significant correlations.

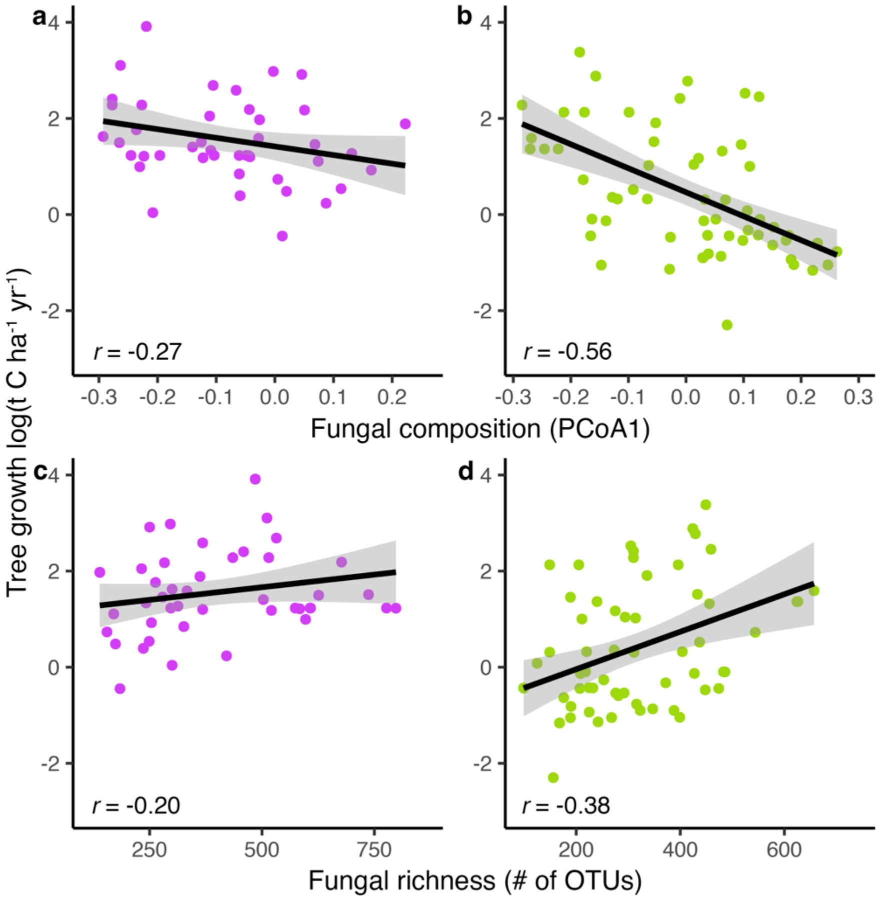

Supplementary Fig. 2. Correlations between tree growth and fungal composition and richness separated by dominant forest tree type. Green and purple points show conifers and broadleaves, respectively.

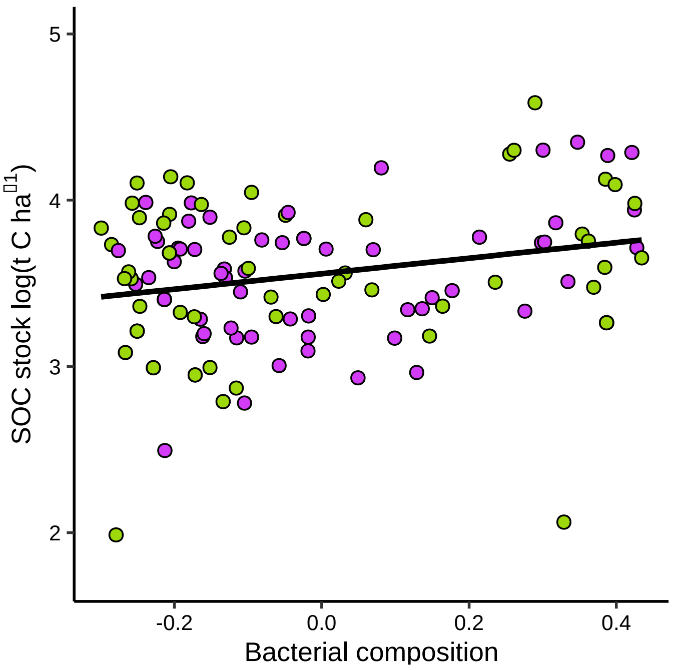

Supplementary Fig. 3. The weak correlation between bacterial community composition (PCoA1) and mineral horizon SOC stocks (r = 0.24, P = 0.006). Only PCoA1 is shown since PCoA2 was not correlated with mineral horizon SOC stocks. Green and purple points show conifers and broadleaves, respectively.

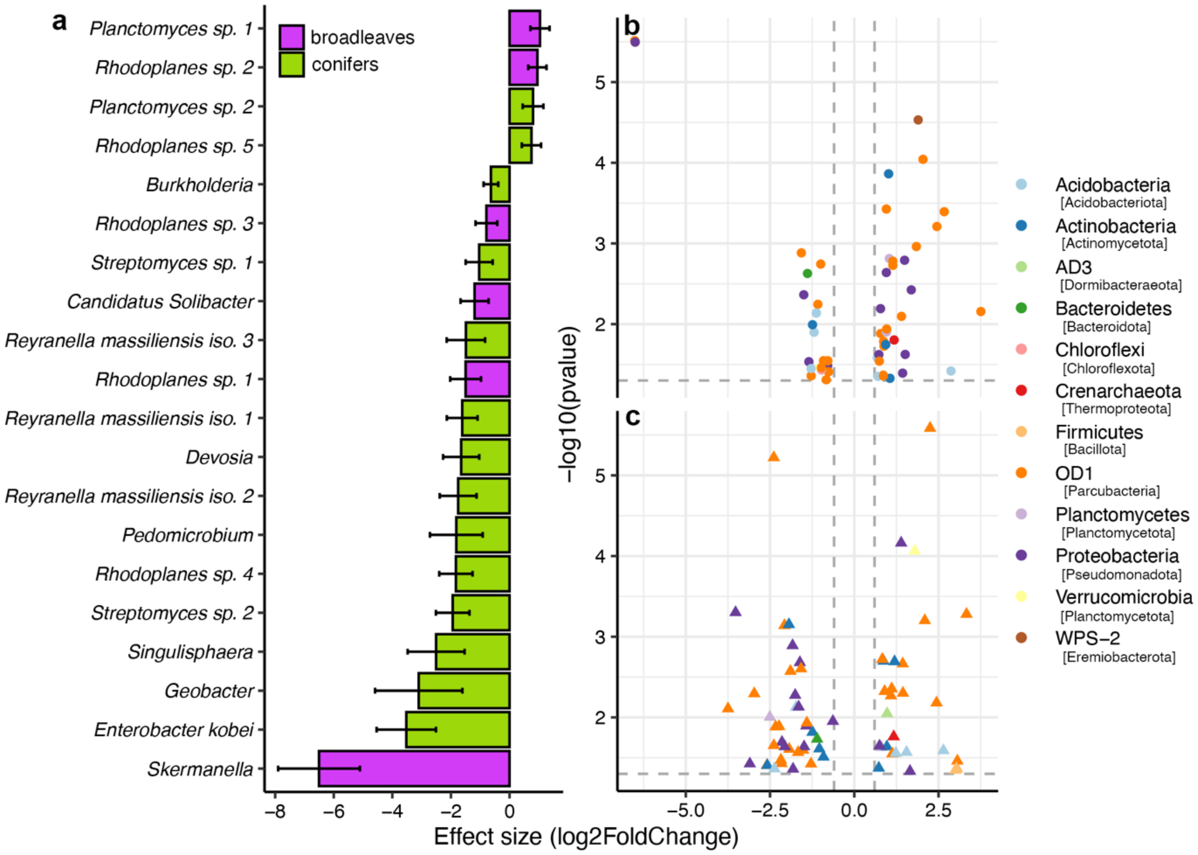

Supplementary Fig. 4. Bacterial taxa linked to variation in organic horizon soil organic carbon stocks. Significant bacterial indicator species identified from DESeq2 analysis summarized from the species to phylum level. Significant indicator species of organic horizon carbon stocks that could be assigned genus and species level taxonomies using reference databases (**a**). Volcano plot showing the strength of all bacterial OTUs significantly correlated to soil carbon stocks in broadleaf (**b**) and conifer (**c**) forests. Values less and more than 0 indicate negative and positive correlations with organic horizon carbon stocks, respectively. Taxonomic labels in square brackets reflect the updated International Committee on Systematics of Prokaryotes (ICSP) phyla names.

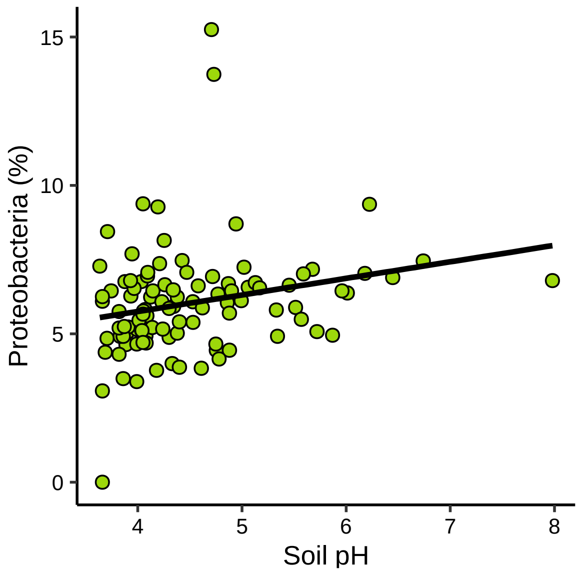

Supplementary Fig. 5. Correlation between Proteobacteria relative abundances and soil pH in the organic horizon of coniferous forests (*r* = 0.24, P = 0.02)

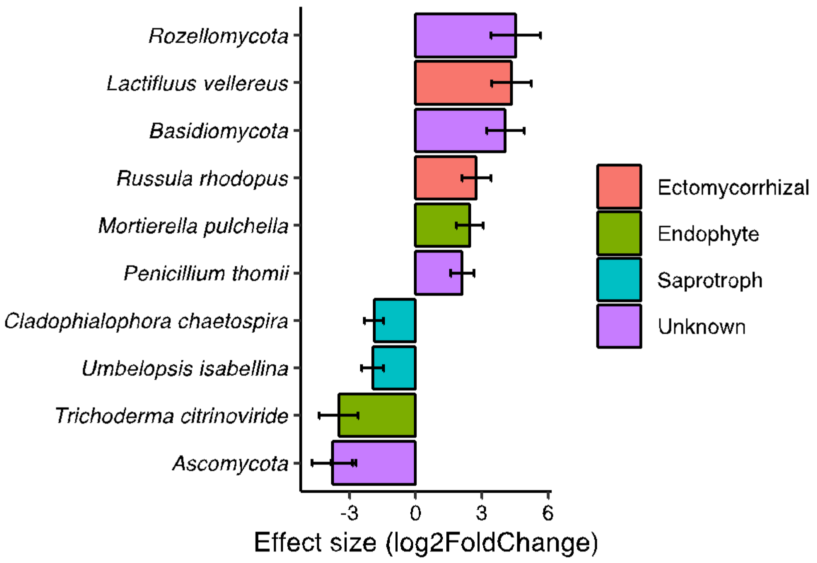

Supplementary Fig. 6. Fungal taxa linked to variation in organic horizon soil organic carbon stocks. Significant fungal indicator species were identified from DESeq2 analysis (see methods for a complete description). Taxa are labelled at the finest taxonomic resolution possible. Values less and more than 0 indicate negative and positive correlations with organic horizon carbon stocks, respectively.

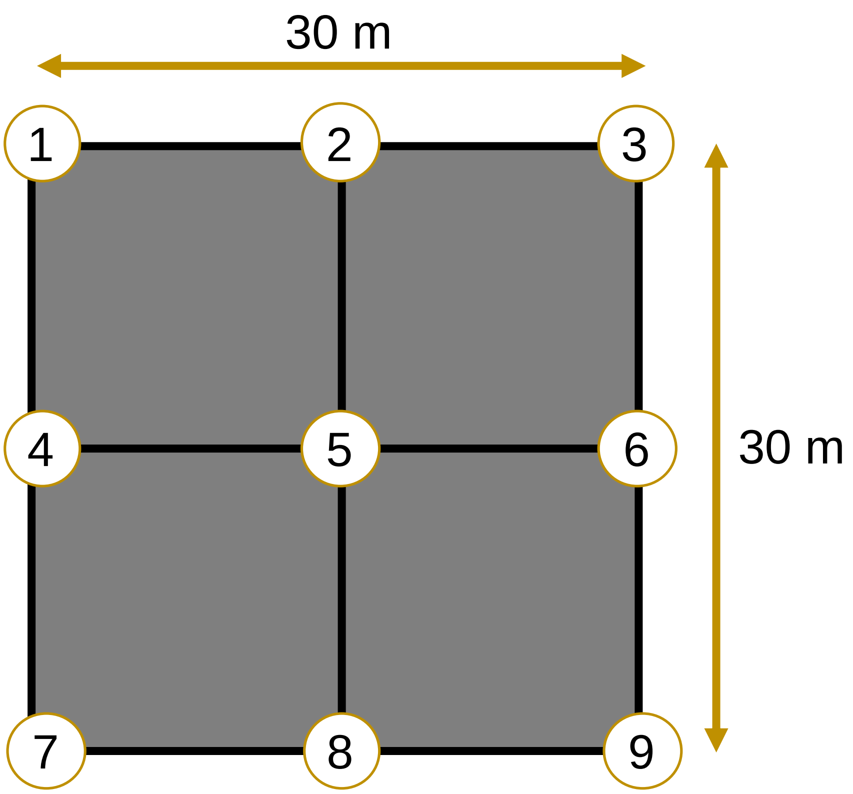

Supplementary Fig. 7. Visualization of the grid-design used for soil sampling. Soil cores were collected at positions 1-9 and pooled into one composite sample per depth increment (organic horizon and mineral soil at a 0-10 cm depth).
