# Supplementary material for: Fungal community composition predicts forest carbon storage at a continental scale
Source: Nat Commun. 2024 Mar 16;15:2385. doi: 10.1038/s41467-024-46792-w (PMC10944544; doi:10.1038/s41467-024-46792-w)
Supplement: Supplementary file 3 — Description of Additional Supplementary Files [file 41467_2024_46792_MOESM3_ESM.pdf]

Description of Additional Supplementary Files

File Name: Supplementary Data 1
Description: Statistical summaries comparing models of tree growth with soil microbiome community composition represented as principal coordinate analysis (PCoA) axes 1 & 2 as predictor variables. Predictors included PCoA axes for total fungi, total bacteria, and different fungal functional groups. Data shows the results of generalized additive modeling, including model estimates (i.e., coefficients), standard errors (SE), and *P*-values from full statistical models including all co-variables. Note that each model contained only one microbiome predictor. Models with 'pure' ectomycorrhizal fungi and 'pure' saprotrophs are those that include fungal OTUs exclusively assigned to one versus multiple functional guilds (e.g., saprotroph-pathogen). Note that pure category models were not made for endophytes or pathogens because it is widely expected that both endophytes and pathogens will have more than one trophic mode. We only show models of tree growtha, not tree biomass, to avoid redundancy because the results are virtually identical for tree biomass (see Fig. 1). Abbreviations: *N = nitrogen; MAT = mean annual temperature; MAP = mean annual precipitation*.

File Name: Supplementary Data 2
Description: Significant ectomycorrhizal and endophytic indicator OTUs of variation in forest tree growth rates. Results are from DESeq2 analysis which estimates variance-mean dependence in relative abundance using a negative binomial distribution. Only OTUs with Log2FoldChanges >0.6 or less than <-0.6 are included, which indicate negative and positive correlations with tree growth rates, respectively (see the methods section for a complete description of this technique). The guild listed is a single assignment made to either the ectomycorrhizal or endophytic guild. Note that it is not necessarily the only guild to which an OTU was assigned. Not all OTUs could be assigned to the species (*spp*) level, and these OTUs are referred to by the genus only or as unidentified if the genus is unknown. Note that models were run separately for conifers and broadleaves, but results are assembled for summary purposes.

File Name: Supplementary Data 3
Description: Statistical summaries comparing models of soil carbon stocks in the organic horizon with fungal versus bacterial community composition represented as principal coordinates analysis (PCoA) axes 1 and 2 and species richness (# of operational taxonomic units; OTUs) as predictor variables. Data shows the results of generalized additive modeling, including model estimates (i.e., coefficients), standard errors (SE), and *P*-values from full statistical models including all co-variables. Note that each model contains only one microbiome predictor.

File Name: Supplementary Data 4
Description: Statistical summaries of soil organic carbon stocks in the organic and mineral soil horizons without microbiome predictors. Data shown is from generalized additive modeling and includes model estimates (i.e. coefficient), standard errors (SE), t-values, and *P*-values. Separate models were made for tree growth and tree biomass because the two variables are strongly correlated and cannot be model together. Note that different smoothing parameters were used for the two soil horizons due to different correlation structures between soil organic carbon stocks and the predictor variables. Abbreviations: *N = nitrogen; MAT = mean annual temperature; MAP = mean annual precipitation*

File Name: Supplementary Data 5
Description: Indicator fungal OTUs of organic horizon SOC stocks in broadleaf forests ordered from most negative to most positive. Results are from DESeq2 analysis which estimates variance-mean dependence in relative abundance using a negative binomial distribution. Only OTUs with Log2FoldChanges >0.6 or less than <-0.6 are included, and these OTUs indicate negative and positive correlations with organic horizon carbon stocks, respectively (see the methods section for a complete description of the technique). FunGuild assignments were abbreviated to shorten the character string: PP = plant pathogen, U = undefined, W = wood, AP = animal pathogen, Total mix = had too many assignments to abbreviate, na = not assigned.

File Name: Supplementary Data 6
Description: Numbers of quality-controlled, 16S (bacteria) and full-length ITS (fungi) sequences prior to rarefication in samples that we included in the analysis because they contained >5,000 and >500 sequences, respectively.

File Name: Supplementary Data 7
Description: Variance inflation factors (VIF) for statistical models of tree growth and organic horizon carbon stocks. VIF scores are derived from generalized additive model results. Note that these two response variables were selected because they were the two responses most tightly coupled to select components of the soil microbiome. Each model contained one microbiome predictor, although results are summarized across all models in the same table. Each time a new principal coordinates analysis (PCoA) axis appears in a row, this denotes the start of a new, independent model. Note that we only show results for PCoA1 for tree growth because it was more tightly linked to tree growth than PCoA2, and this is what was used for visualizations and comparative analyses in the manuscript. Conversely, we show PCoA2 for organic horizon carbon stocks for the same reason.
