# Supplementary material for: Fungal community composition predicts forest carbon storage at a continental scale
Source: Nat Commun. 2024 Mar 16;15:2385. doi: 10.1038/s41467-024-46792-w (PMC10944544; doi:10.1038/s41467-024-46792-w)
Supplement: Supplementary file 5 — Reporting Summary [file 41467_2024_46792_MOESM5_ESM.pdf]

nature portfolio

Corresponding author(s): Mark Anthony
Last updated by author(s): 2024.15.2

Reporting Summary

Nature Portfolio wishes to improve the reproducibility of the work that we publish. This form provides structure for consistency and transparency in reporting. For further information on Nature Portfolio policies, see our Editorial Policies and the Editorial Policy Checklist.

Statistics

For all statistical analyses, confirm that the following items are present in the figure legend, table legend, main text, or Methods section.

n/a	Confirmed	
☐	☒	The exact sample size (*n*) for each experimental group/condition, given as a discrete number and unit of measurement
☐	☒	A statement on whether measurements were taken from distinct samples or whether the same sample was measured repeatedly
☐	☒	The statistical test(s) used AND whether they are one- or two-sided<br>*Only common tests should be described solely by name; describe more complex techniques in the Methods section.*
☐	☒	A description of all covariates tested
☐	☒	A description of any assumptions or corrections, such as tests of normality and adjustment for multiple comparisons
☐	☒	A full description of the statistical parameters including central tendency (e.g. means) or other basic estimates (e.g. regression coefficient) AND variation (e.g. standard deviation) or associated estimates of uncertainty (e.g. confidence intervals)
☐	☒	For null hypothesis testing, the test statistic (e.g. *F*, *t*, *r*) with confidence intervals, effect sizes, degrees of freedom and *P* value noted<br>*Give P values as exact values whenever suitable.*
☒	☐	For Bayesian analysis, information on the choice of priors and Markov chain Monte Carlo settings
☒	☐	For hierarchical and complex designs, identification of the appropriate level for tests and full reporting of outcomes
☐	☒	Estimates of effect sizes (e.g. Cohen's *d*, Pearson's *r*), indicating how they were calculated

Our web collection on statistics for biologists contains articles on many of the points above.

Software and code

Policy information about availability of computer code

Data collection	All scripts used for the analysis are openly available at the following repository: https://gitlab.com/fungalecology/icpf.micro.
Data analysis	QIIME2 (v2021.8), ITSx (v1.1.3), UNITE (v8, 2021-10 release), Greengenes (2019-05 release), vegan package in R (v2.6-4), ape package in R (5.6-2), DESeq2 package in R (1.34.0), mgcv package in R (1.8-38)

For manuscripts utilizing custom algorithms or software that are central to the research but not yet described in published literature, software must be made available to editors and reviewers. We strongly encourage code deposition in a community repository (e.g. GitHub). See the Nature Portfolio guidelines for submitting code & software for further information.

Data

Policy information about availability of data

All manuscripts must include a data availability statement. This statement should provide the following information, where applicable:

- Accession codes, unique identifiers, or web links for publicly available datasets
- A description of any restrictions on data availability
- For clinical datasets or third party data, please ensure that the statement adheres to our policy

Full access to raw ICP Forest datasets is available via the ICP Forests network upon request (http://icp-forests.net/page/data-requests). Restrictions apply to the availability of these data without a formal data request. Raw microbiome datasets can be downloaded from the NCBI SRA (https://www.ncbi.nlm.nih.gov/sra) using accession numbers PRJNA1068067, PRJNA639984, PRJNA644776, and PRJNA1068308. Microbiome and other data products can be downloaded in the following

repository https://gitlab.com/fungalecology/icpf.micro.The fungal taxonomic database UNITE can be accessed here: https://unite.ut.ee/index.php while the bacterial taxonomic database Greengenes can be accessed here: https://greengenes.secondgenome.com/. Fungal functional group data from FUNGuild can be accessed here: http://www.funguild.org/.

Research involving human participants, their data, or biological material

Policy information about studies with human participants or human data. See also policy information about sex, gender (identity/presentation), and sexual orientation and race, ethnicity and racism.

Reporting on sex and gender	NA
Reporting on race, ethnicity, or other socially relevant groupings	NA
Population characteristics	NA
Recruitment	NA
Ethics oversight	NA

Note that full information on the approval of the study protocol must also be provided in the manuscript.

Field-specific reporting

Please select the one below that is the best fit for your research. If you are not sure, read the appropriate sections before making your selection.

☐ Life sciences ☐ Behavioural & social sciences ☒ Ecological, evolutionary & environmental sciences

For a reference copy of the document with all sections, see nature.com/documents/nr-reporting-summary-flat.pdf

Life sciences study design

All studies must disclose on these points even when the disclosure is negative.

Sample size	*Describe how sample size was determined, detailing any statistical methods used to predetermine sample size OR if no sample-size calculation was performed, describe how sample sizes were chosen and provide a rationale for why these sample sizes are sufficient.*
Data exclusions	*Describe any data exclusions. If no data were excluded from the analyses, state so OR if data were excluded, describe the exclusions and the rationale behind them, indicating whether exclusion criteria were pre-established.*
Replication	*Describe the measures taken to verify the reproducibility of the experimental findings. If all attempts at replication were successful, confirm this OR if there are any findings that were not replicated or cannot be reproduced, note this and describe why.*
Randomization	*Describe how samples/organisms/participants were allocated into experimental groups. If allocation was not random, describe how covariates were controlled OR if this is not relevant to your study, explain why.*
Blinding	*Describe whether the investigators were blinded to group allocation during data collection and/or analysis. If blinding was not possible, describe why OR explain why blinding was not relevant to your study.*

Behavioural & social sciences study design

All studies must disclose on these points even when the disclosure is negative.

Study description	*Briefly describe the study type including whether data are quantitative, qualitative, or mixed-methods (e.g. qualitative cross-sectional, quantitative experimental, mixed-methods case study).*
Research sample	*State the research sample (e.g. Harvard university undergraduates, villagers in rural India) and provide relevant demographic information (e.g. age, sex) and indicate whether the sample is representative. Provide a rationale for the study sample chosen. For studies involving existing datasets, please describe the dataset and source.*
Sampling strategy	*Describe the sampling procedure (e.g. random, snowball, stratified, convenience). Describe the statistical methods that were used to predetermine sample size OR if no sample-size calculation was performed, describe how sample sizes were chosen and provide a rationale for why these sample sizes are sufficient. For qualitative data, please indicate whether data saturation was considered, and what criteria were used to decide that no further sampling was needed.*
Data collection	*Provide details about the data collection procedure, including the instruments or devices used to record the data (e.g. pen and paper, computer, eye tracker, video or audio equipment) whether anyone was present besides the participant(s) and the researcher, and*

	whether the researcher was blind to experimental condition and/or the study hypothesis during data collection.
Timing	*Indicate the start and stop dates of data collection. If there is a gap between collection periods, state the dates for each sample cohort.*
Data exclusions	*If no data were excluded from the analyses, state so OR if data were excluded, provide the exact number of exclusions and the rationale behind them, indicating whether exclusion criteria were pre-established.*
Non-participation	*State how many participants dropped out/declined participation and the reason(s) given OR provide response rate OR state that no participants dropped out/declined participation.*
Randomization	*If participants were not allocated into experimental groups, state so OR describe how participants were allocated to groups, and if allocation was not random, describe how covariates were controlled.*

Ecological, evolutionary & environmental sciences study design

All studies must disclose on these points even when the disclosure is negative.

Study description	A soil microbiome study collected across 285 forest monitoring plots. At each site, nine soil samples were collected in a grid design (see Supplementary Fig. 7) along a 30 x 30 m grid and pooled into a single sample per site.
Research sample	The samples are soils, collecting from the forest floor or at a 0-10 cm depth for mineral soil.
Sampling strategy	Sample sizes were selected to maximize coverage across across Europe and for feasibility for the work to be conducted by one postdoctoral researcher. At each site, the forest floor (i.e. organic horizon) was removed as a 5 x 5 cm block using a serrated knife and spatula. After removing the organic horizon, a soil core (5 cm wide x 10 cm deep) was taken immediately beneath the same area. This procedure was repeated 9 times along the 30 x 30 m grid, and all samples were pooled together by depth increment into a single composite sample per depth increment. Samples were then dried in a drying oven set to 40 degrees C max for 24 hours, on a heated floor, in a closed dry room under sunlight, under a windshield of a car in sunlight, outside in the sun, or above a fireplace, stove, or beside a fire until the samples were completely dried.
Data collection	Data was collected by the authors or their research technicians in July and August in 2019 and 2020
Timing and spatial scale	July, 2019-August, 2020, Europe.
Data exclusions	Samples with low sequencing depth were removed from the dataset (<5,000 sequences for 16S analysis; <500 sequences for ITS analysis). Samples with incomplete co-variables were excluded from generalized additive models because complete observations are required. We removed any dead trees, trees with <5 cm DBH, trees that shrank over the growth period from our calculations of tree growth because they are not reaching quality control standards or represent measurement errors.
Reproducibility	We used established, best practices for sampling protocols in the field and in the laboratory so our results can be compared to other work and reproduced.
Randomization	Genomic DNA was randomly extracted from samples in no particular order.
Blinding	Samples were dummy labeled in a random numeric order and each sample was therefore blind during the PCR and laboratory work.

Did the study involve field work? ☒ Yes ☐ No

Field work, collection and transport

Field conditions	Samples were collected in forests in the summer time under heterogenous conditions.
Location	Country (latitude, longitude)<br>France (48.2, 4.18)<br>France (46.1, 5.14)<br>France (47.56, 7.07)<br>France (48.17, 4.27)<br>France (47.04, 5.04)<br>France (48.1, -1.32)<br>France (47.34, 1.15)<br>France (49.02, 4.57)<br>France (46.49, 2.34)<br>France (49, 7.27)<br>France (47.47, 0.22)<br>France (44.02, 1.44)<br>France (46.37, 0.29)<br>France (48.59, 7.43)

France (43.44, -0.5)
France (48.27, 2.43)
France (43.26, 2.42)
France (48.34, -0.04)
France (45.56, 4.28)
France (49.56, 4.48)
France (43.38, 2.55)
France (47.27, -0.01)
France (46.31, 6.03)
France (43.26, 2.26)
France (49.12, 3.07)
France (46.11, 2.59)
France (44.07, 5.48)
France (42.55, 1.16)
France (47.48, 4.51)
France (48.3, 6.42)
France (48.38, 6.04)
France (43.09, -0.39)
France (43.01, 0.26)
France (43.24, 2.1)
France (44.42, 6.33)
France (42.15, 8.5)
France (43.12, -0.02)
France (47.39, 2.05)
France (47.44, 0.2)
France (44.51, 3.03)
France (47.39, 2.05)
France (47.52, 6.12)
France (48.36, -0.3)
France (45.24, 3.41)
France (49.27, 0.44)
France (48.41, 1.43)
France (47.56, 3.43)
France (46.58, 5.14)
France (46.58, 6.27)
France (45.25, 6.07)
Spain (40.11, -6.26)
Spain (40.19, -0.43)
Spain (38.31, -0.37)
Spain (41.52, -2.53)
Spain (42.51, -4.33)
Spain (41.21, -4.17)
Spain (40.32, 0.01)
Spain (39.56, -5.47)
Finland (60.38, 23.48)
Finland (60.36, 23.5)
Finland (61.14, 25.04)
Finland (63.09, 30.42)
Finland (67.59, 24.14)
Belgium (51.04, 3.02)
Belgium (51.24, 5.03)
Belgium (51.18, 4.31)
Belgium (50.58, 3.48)
Belgium (50.44, 4.24)
Germany (54.06, 10.14)
Germany (52.28, 13.13)
Germany (53.08, 12.57)
Germany (52.58, 13.38)
Germany (52.11, 12.33)
Germany (51.47, 13.33)
Germany (52.09, 14.29)
Germany (52.34, 11.1)
Germany (52.03, 12.18)
Germany (52.34, 11.11)
Germany (52.5, 10.15)
Germany (51.45, 9.34)
Germany (50.09, 9.23)
Germany (51.22, 9.16)
Germany (51.09, 9.01)
Germany (49.38, 8.5)
Germany (49.42, 8.32)
Germany (49.41, 7.01)
Germany (49.16, 7.48)
Germany (49.18, 7.51)
Germany (49.24, 11.19)
Germany (48.13, 12.44)

Germany (50.21, 9.55)
Germany (49.07, 10.35)
Germany (49.51, 10.31)
Germany (48.58, 12.52)
Germany (48.55, 11.45)
Germany (49.58, 9.27)
Germany (47.34, 10.23)
Germany (48.24, 11.39)
Germany (49.43, 9.53)
Germany (47.43, 11.4)
Germany (48.17, 11.04)
Italy (43.17, 13.04)
Italy (45.4, 8.04)
Switzerland (46.39, 10.13)
Switzerland (47.41, 8.32)
Switzerland (47.24, 8.13)
Switzerland (46.17, 7.51)
Switzerland (47.16, 7.53)
Switzerland (47.09, 9.04)
Switzerland (46.49, 9.51)
Switzerland (47.28, 8.21)
Switzerland (46.42, 7.45)
Switzerland (47.13, 7.25)
Switzerland (46.29, 9.53)
Switzerland (46.26, 8.48)
Switzerland (46.07, 9)
Switzerland (46.13, 6.17)
Switzerland (46.35, 6.39)
Switzerland (46.16, 7.26)
Poland (53.53, 14.3)
Poland (54.12, 17.13)
Poland (54.33, 18.16)
Poland (53.59, 19.26)
Poland (52.4, 23.43)
Poland (53.41, 16.46)
Poland (53.49, 17.51)
Poland (52.57, 18.31)
Poland (52.2, 22.39)
Poland (51.58, 22.59)
Poland (51.29, 23.21)
Poland (51.1, 21.52)
Poland (50.3, 23.28)
Poland (50.16, 22.07)
Poland (50.5, 15.25)
Poland (51.03, 15.52)
Poland (49.39, 22.29)
Poland (49.27, 22.17)
Poland (49.29, 21.37)
Norway (58.23, 8.14)
Norway (60.22, 11.04)
Norway (61.16, 11.51)
Lithuania (55.49, 21.28)
Lithuania (54.51, 23.32)
Estonia (59.33, 26.02)
Estonia (59.34, 26.07)
Estonia (58.03, 26.06)
Estonia (57.42, 26.31)
Estonia (59.31, 26.24)
Estonia (58.16, 26.27)
Slovenia (46.29, 16.31)
Slovenia (46.27, 15.23)
Slovenia (46.17, 14.24)
Slovenia (45.32, 14.48)
Slovenia (46.06, 15.04)
Bulgaria (42.46, 23.45)
Bulgaria (43.03, 27.46)
Bulgaria (42.04, 23.48)
Bulgaria (41.41, 24.44)
Bulgaria (42.54, 27.45)
Latvia (57.1, 25.41)
Latvia (56.44, 23.41)
Denmark (55.22, 11.33)
Denmark (55.42, 12.21)
France (46.4, 2.43)
France (47.15, 2.07)
France (49.21, 1.3)

France (48.52, 6.29)
France (48.31, 0.4)
France (47.41, 7.28)
France (46.09, 1.49)
France (43.06, -0.06)
France (45.45, 2.57)
France (45.35, 6.47)
France (46.13, 6.2)
France (45.48, 1.48)
France (49.1, -0.51)
France (44.55, 5.17)
France (49.1, 5)
France (49.42, 1.19)
France (48.06, 6.14)
France (41.45, 9.12)
France (43.56, -1.2)
France (44.02, 0)
France (44.01, 6.4)
France (48.12, -1.33)
France (47.49, 2.26)
France (48.51, 7.42)
France (48.55, 7.26)
France (44.29, 6.27)
France (44.42, 3.57)
France (42.51, 1.2)
France (44.56, 5.19)
France (46.5, 5.47)
France (48.36, 7.08)
Spain (42.44, -8.42)
Spain (42.59, -1.2)
Spain (38.1, -4.04)
Spain (40.52, -3.58)
Finland (69.34, 28.53)
Germany (51.45, 9.34)
Germany (51.31, 10.02)
Germany (52.54, 7.51)
Germany (50.41, 8.38)
Germany (48.07, 11.55)
Switzerland (46.01, 8.5)
Poland (54.16, 22.5)
Poland (53.4, 21.26)
Poland (52.51, 23.4)
Poland (51.46, 17.34)
Poland (53.07, 19.47)
Poland (52.43, 20.36)
Poland (52.01, 21.06)
Poland (51.48, 21.27)
Poland (52.46, 22.4)
Poland (51.39, 21.02)
Poland (50.3, 22.56)
Poland (49.2, 20.5)
Poland (49.27, 19.59)
Slovenia (45.52, 15.25)
Bulgaria (42.03, 23.55)

Access & import/export	All soil import was legal and reported. Soil importation (permit Nr. 26/20) was granted by the Bundesamt für Landwirtschaft BLW from March 16 2019-March 15 2021.
Disturbance	None

Reporting for specific materials, systems and methods

We require information from authors about some types of materials, experimental systems and methods used in many studies. Here, indicate whether each material, system or method listed is relevant to your study. If you are not sure if a list item applies to your research, read the appropriate section before selecting a response.

Materials & experimental systems

n/a	Involved in the study
☒	☐ Antibodies
☒	☐ Eukaryotic cell lines
☒	☐ Palaeontology and archaeology
☒	☐ Animals and other organisms
☒	☐ Clinical data
☒	☐ Dual use research of concern
☒	☐ Plants

Methods

n/a	Involved in the study
☒	☐ ChIP-seq
☒	☐ Flow cytometry
☒	☐ MRI-based neuroimaging

Plants

Seed stocks	*Report on the source of all seed stocks or other plant material used. If applicable, state the seed stock centre and catalogue number. If plant specimens were collected from the field, describe the collection location, date and sampling procedures.*
Novel plant genotypes	*Describe the methods by which all novel plant genotypes were produced. This includes those generated by transgenic approaches, gene editing, chemical/radiation-based mutagenesis and hybridization. For transgenic lines, describe the transformation method, the number of independent lines analyzed and the generation upon which experiments were performed. For gene-edited lines, describe the editor used, the endogenous sequence targeted for editing, the targeting guide RNA sequence (if applicable) and how the editor was applied.*
Authentication	*Describe any authentication procedures for each seed stock used or novel genotype generated. Describe any experiments used to assess the effect of a mutation and, where applicable, how potential secondary effects (e.g. second site T-DNA insertions, mosiacism, off-target gene editing) were examined.*
